# Moyamoya Disease in a 13-Month-Old Middle Eastern Boy

**DOI:** 10.7759/cureus.18874

**Published:** 2021-10-18

**Authors:** Nashwa M. Ali, Abdullah Alawad, Ahmed Alferayan, Ahmed Al-Rumayyan, Sawsan Alkoury

**Affiliations:** 1 College of Medicine, Alfaisal University, Riyadh, SAU; 2 Interventional Radiology, King Saud Medical City, Riyadh, SAU; 3 Neurosurgery, Specialized Medical Center, Riyadh, SAU; 4 Neurology, King Saud Bin Abdulaziz University for Health Sciences, Riyadh, SAU; 5 Pediatrics, Specialized Medical Center, Riyadh, SAU

**Keywords:** moyamoya disease (mmd), seizure, moyamoya syndrome, encephaloduroarteriosynangiosis (edas), puff of smoke, mri

## Abstract

Moyamoya disease (MMD) is a rare, chronic cerebrovascular disease affecting the cerebral arteries, leading to the development of unique collateral vessels. Few cases were reported from Saudi Arabia; however, the incidence rate is not well-defined. Hence, we present a case of a 13-month-old child who presented to the emergency room with first onset focal seizures with relatively unremarkable past medical and family history. Investigations were ordered accordingly including head computed tomography (CT) scan, brain magnetic resonance imaging (MRI), and cerebral angiogram, and he was diagnosed with MMD and considering a broader variety of differential diagnoses for seizures in children is highlighted in our case. Furthermore, considering their predominance in east Asian countries, it highlights a rare presentation in the middle eastern race.

## Introduction

Moyamoya disease (MMD) is a rare, chronic cerebrovascular disorder that is characterized by progressive stenosis of the intracranial internal carotid artery (ICA) and its main branches with the formation of an abnormal vascular network or collaterals at the base of the brain [1]. Imaging of the collaterals resembles a “puff of smoke,” or moyamoya in Japanese [2]. The incidence of MMD exhibits significant regional differences, with the highest incidence in East Asia [3]. According to prior research, the prevalence of MMD in Japan is 10.5 per 100,000 individuals, with an incidence rate of 0.94 per 100,000 individuals [4]. Data from a nationwide registry in Japan, with 2,545 cases of MMD, showed a bimodal age of onset, with one peak at approximately 10 years of age and adults presenting at approximately 40 years of age. However, presentation in infancy is uncommon [5].

The most common initial presentation of moyamoya is ischemic stroke and recurrent transient ischemic attacks (TIAs). Patients may infrequently present with seizures due to underlying ischemic damage, and the rate of seizures may be more frequent in children than in adults [5]. Cerebral ischemia and cerebral hemorrhage are the most common clinical signs of MMD. The distribution of these two types of symptoms differs between children and adults. The majority of pediatric patients have progressive cerebral ischemia which can include TIAs and cerebral infarctions. In infants, mental derangement or seizures may be initial presentation [3].

Definitive diagnosis of MMD or syndrome is based on the following conventional angiographic criteria: (1) Stenosis of the terminal portion of the intracranial ICA or proximal portions of the anterior cerebral artery (ACA) and/or the middle cerebral artery (MCA). (2) Development of vascular collaterals near the stenotic lesions in the arterial phase. (3) Bilateral lesion. After the Research Committee on Spontaneous Occlusion of the Circle of Willis (MMD) in Japan revised the diagnostic criteria in 1995, definitive diagnosis of MMD has also been made by meeting all of the following items by MR imaging/angiography without conventional catheter angiography: (1) MR angiography (MRA) shows stenosis or occlusion of the intracranial ICA's terminal segment, as well as the ACA and/or MCA's proximal segments. (2) MRA showing two or more flow voids in the basal ganglia on each hemisphere, or MR imaging indicating abnormal vascular networks near occlusive or stenotic lesions [6].

Finally, comparable cerebrovascular lesions linked to the following underlying diseases must be ruled out: atherosclerosis, autoimmune disorders, meningitis, brain tumors, Down syndrome, neurofibromatosis type-1, traumatic brain injury, cranial irradiation, and so on [6].

In Saudi Arabia, MMD has rarely been reported and roughly around 20 scattered cases have been reported [7]. Herein, we report a case of MMD in a 13-month-old child who presented with sudden onset of focal seizures.

## Case presentation

A 13-month-old Syrian baby boy born to a non-consanguineous couple was admitted to our hospital with complaints of sudden onset focal tonic-clonic seizure, mainly on the right side, for further evaluation and management. The seizure attack lasted approximately a few minutes. The second attack was associated with loss of consciousness and uprolling of the eyes. There was a history of sudden falls during playing. However, there was no history of fever, head trauma, headache, vomiting, or ear discharge. He had normal growth parameters. there was no significant family history of early stroke, ischemic heart disease, or autoimmune disease. His past medical history was significant for right arm weakness one month before his presentation and resolved without treatment. Upon presentation, he was vitally normal except for HR of 128, and RR of 28.

A head CT revealed nonspecific hypodensity, bilateral periventricular white matter, and the left parietofrontal subcortical area requiring further workup.

**Figure 1:**
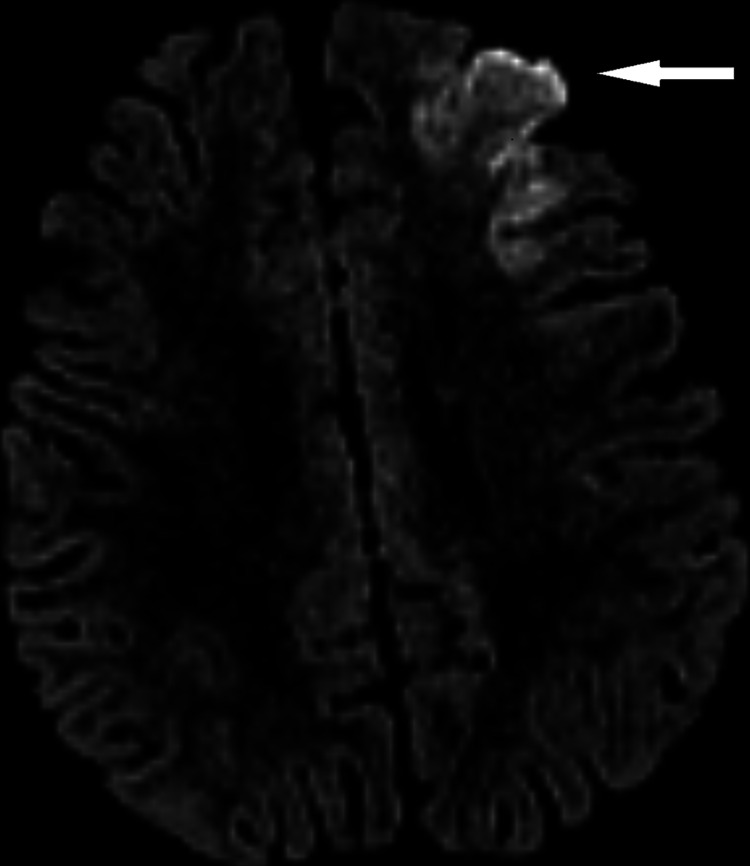
MRI shows left frontal cortical and subcortical area of restricted diffusion consistent with acute infarct.

**Figure 2:**
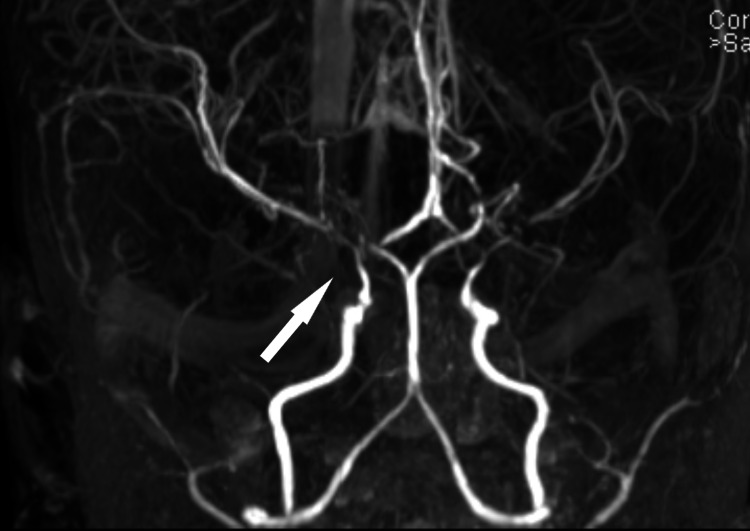
Attenuated and irregular distal intracranial ICAs at the cavernous and supraclinoid segments (MRA). Bilateral stenosis is seen at both MCAs, almost occluded on the right side, and are surrounded by extensive lenticulostriate collaterals. ICA - internal carotid artery; MCA - middle cerebral artery

An MRI (Figure 1) revealed acute infarction involving the left frontal lobe cortical and subcortical region and multiple bilateral periventricular white matter changes likely due to leu-co-encephalopathy/chronic ischemic. MRA (Figure 2) showed attenuated and irregular distal intracranial ICAs at the cavernous and supraclinoid segments and bilateral stenosis seen at both MCAs, almost occluded on the right side, and are surrounded by extensive lenticulostriate collaterals.

Encephalo-duro-arterio-synangiosis (EDAS) surgery was performed due to failure of the first attempt of cerebral angiography. The baby was discharged home in a stable condition on Tegretol and aspirin for follow-up in the clinic and revascularization surgery on the right side was scheduled three months after the first one.

Three weeks later, the child presented with lethargy, weakness in his left arm, and vomiting. Upon admission, he was afebrile, maintaining room air. Physical examination findings were normal apart from bilateral chest rhonchi, and weakness of the left upper and lower limbs. Hematologists recommended starting the patient on clexane and continuing with aspirin.

**Figure 3:**
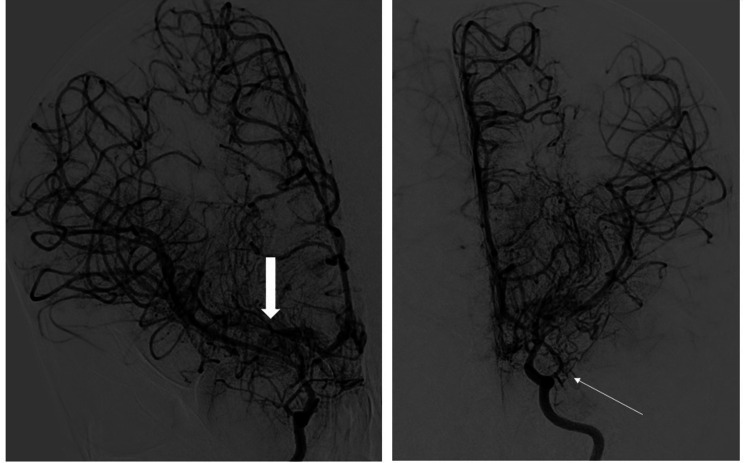
Conventional angiography demonstrates bilateral internal carotid arteries stenosis seen at the carotid terminus with multiple collateral formation compatible with a history of moyamoya disease (left). There is evidence of right middle cerebral artery M2 segment thrombosis compatible with MRI finding of acute infarction. There is evidence of left distal middle cerebral artery branches thrombosis (right).

Conventional cerebral angiography (Figure 3) was performed post left EDAS surgery and demonstrated bilateral ICAs stenosis seen at the carotid terminus with multiple collateral formation compatible with the history of MMD. There is evidence of right MCA M2 segment thrombosis compatible with MRI finding of acute infarction, left distal MCA branches thrombosis, and posterior circulation collateral formation synangiosis to the anterior circulation. Neurologists were consulted, and right-side revascularization was scheduled.

**Figure 4:**
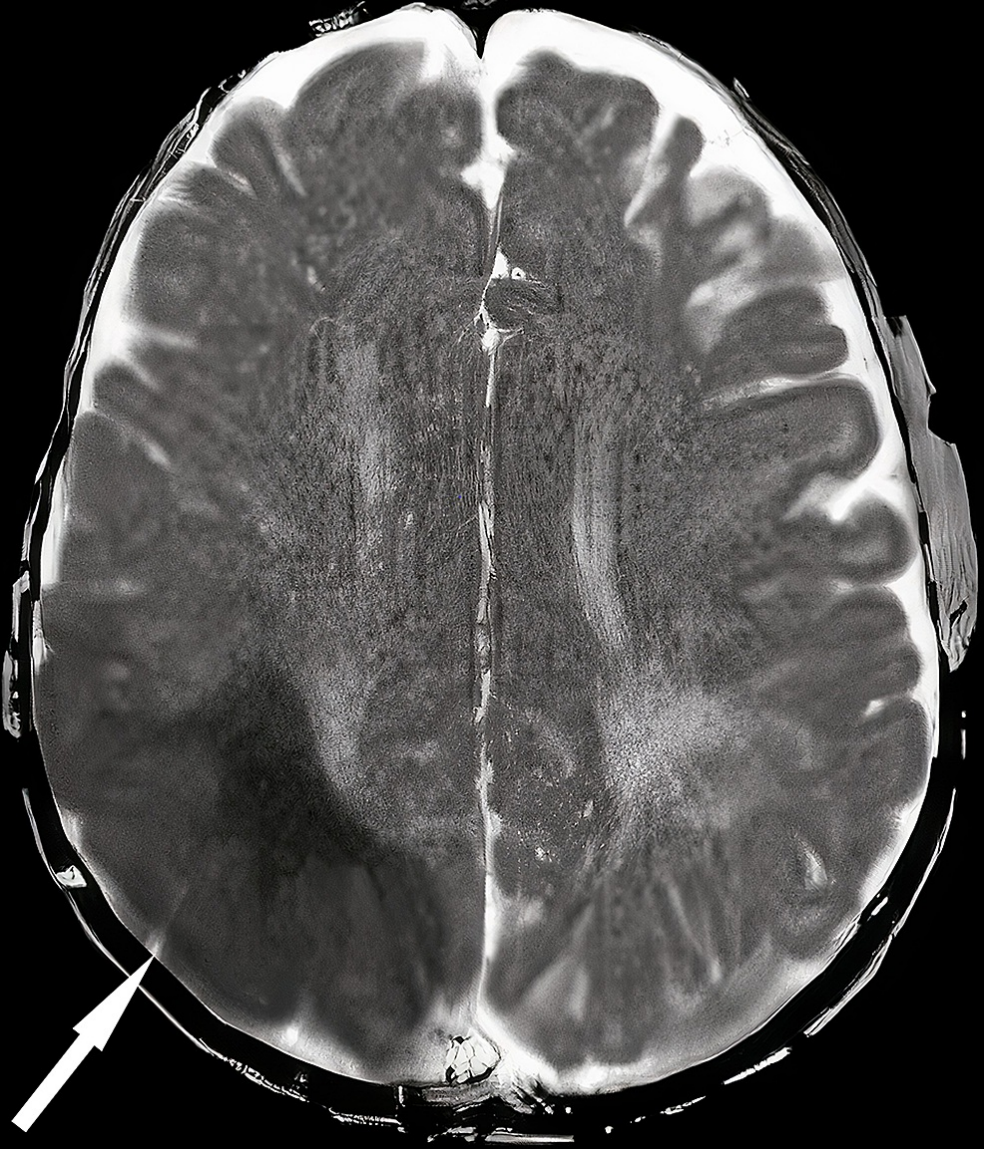
MRI shows large right parietal-temporal-occipital area cortical and subcortical area of restricted diffusion consistent with acute infarct.

A post-operative MRI brain (Figure 4) was performed because the patient still had upper and lower left side weakness, and it revealed a large right parietal-temporal-occipital area cortical and sub-cortical area of restricted diffusion consistent with acute infarct. The previously seen large right cerebral diffusion restriction now shows residual small areas of cortical and adjacent subcortical spaces.

The clinical and radiological findings were suggestive of a progressive vascular occlusive disease that could be seen in MMD. Several investigations including factor V, factor VII, antithrombin activity, protein C, protein S, and EEG were carried to rule out associated medical conditions. The patient was discharged in a stable condition with residual left side weakness. Therefore, an appointment with physiotherapy was reserved.

## Discussion

MMD is a progressive vasculopathy of ICA. Subsequently, compensatory collaterals are formed gradually resembling "puff of smokes," hence the name moyamoya [2]. Females are twice as likely to develop the disease. Herein, we present a case of a male patient. MMD, although difficult to diagnose, its typical presentation and characteristic angiographic findings may aid in its diagnosis [2].

MMD is more common in children and rarely occurs in adults. MMD has a bimodal age of presentation. Children present at around five years of age with clinical symptoms of brain ischemia. Additionally, adults present at around 40 years with hemorrhagic symptoms due to rupture of fragile vessels. Children with MMD usually have recurrent TIAs or infarction in the territory of the ICA, particularly in the frontal lobe, as opposed to adults who usually have subarachnoid or intraparenchymal hemorrhage [8].

Additionally, MMD has an increasing incidence in East Asian countries, particularly Japan and Korea. The occurrence rate in Saudi Arabia is not yet determined. To the best of our knowledge, less than 200 cases have been reported worldwide, and roughly less than 20 cases from Saudi Arabia [9].

The reported incidence of pediatric strokes ranges from 1.2 to 13 cases per 100,000 children under the age of 18; however, recent studies suggest that the incidence is rising [10]. MMD is considered to be one of the leading causes of stroke in this very young age group [7]. Because of the high prevalence of cerebral infarction in young pediatric MMD patients, particularly those younger than four years of age, the prognosis is poor [1]. The high frequency of steno-occlusive lesions of the PCA, poor development of transdural collaterals, and a relatively insufficient blood supply to the developing brain are all potential mechanisms underlying cerebral infarction in patients diagnosed before the age of four [1].

MMD primary treatment is surgical revascularization, without which around two-third of the patients will have poor outcomes [2]. Direct bypassing, which involves anastomosis of the superficial temporal artery to the MCA (STA-MCA), indirect bypassing, which includes encephaloduroarteriosynangiosis (EDAS) and encephalomyosynangiosis (EMS), and combined bypassing are the three forms of surgical procedures [8]. EDAS is most commonly carried in the pediatric age group. In a study of patients with MMD, Duan et al. [6] found that the risk of recurrent cerebral ischemia or hemorrhage in patients who underwent surgical revascularization was significantly lower than in patients who received conservative care.

## Conclusions

Our case brings forth the importance of having a high index of suspicion and considering a wider range of differential diagnoses when managing pediatric patients with seizures. Furthermore, it highlights a rare presentation in Asian Caucasian/middle eastern race, despite the predominance in east Asian countries. Early diagnosis and prompt intervention will improve the prognosis and life expectancy of pediatric MMD patients. The gold standard for diagnosing and making surgical decisions in MMD is conventional cerebral angiography. There is currently no standardized surgical approach for the treatment of MMD in children, and numerous revascularization procedures have been used with relatively good results in hindering disease progression.
